# B cell activation and proliferation increase intracellular zinc levels^☆^

**DOI:** 10.1016/j.jnutbio.2018.10.008

**Published:** 2019-02

**Authors:** Johanna Ollig, Veronika Kloubert, Kathryn M. Taylor, Lothar Rink

**Affiliations:** a Institute of Immunology, Faculty of Medicine, RWTH Aachen University Hospital, Pauwelsstr. 30, 52074 Aachen, Germany; b Breast Cancer Molecular Pharmacology, Welsh School of Pharmacy and Pharmaceutical Sciences, Cardiff University, King Edward VII Avenue, Cardiff, CF10 3NB, United Kingdom

**Keywords:** B Cell, Zinc, Zinc Transporter, ZIP7, phoshoZIP7, proliferation

## Abstract

Zinc ions serve as second messengers in major cellular pathways, including the regulation pathways of proliferation and their proper regulation is necessary for homeostasis and a healthy organism. Accordingly, expression of zinc transporters can be altered in various cancer cell lines and is often involved in producing elevated intracellular zinc levels. In this study, human B cells were infected with Epstein–Barr virus (EBV) to generate immortalized cells, which revealed traits of tumor cells, such as high proliferation rates and an extended lifespan. These cells showed differentially altered zinc transporter expression with ZIP7 RNA and protein expression being especially increased as well as a corresponding increased phosphorylation of ZIP7 in EBV-transformed B cells. Accordingly, free zinc levels were elevated within these cells. To prove whether the observed changes resulted from immortalization or rather high proliferation, free zinc levels in *in vitro* activated B cells and in freshly isolated B cells expressing the activation marker CD69 were determined. Here, comparatively increased zinc levels were found, suggesting that activation and proliferation, but not immortalization, act as crucial factors for the elevation of intracellular free zinc.

## Graphical abstract

Neoplastic transformation of B cells by Epstein–Barr-Virus transformation increased the intracellular free zinc. However, this is not a part of neoplastic transformation, but a physiological response to B cell activation, since B cells activated by CD40 ligand *in vitro*, as well as *in vivo* activated B cells from peripheral blood, indicated by CD69 expression, increased intracellular free zinc. This seems to be mediated by phosphorylation of zinc transporter ZIP7.

**Figure:**
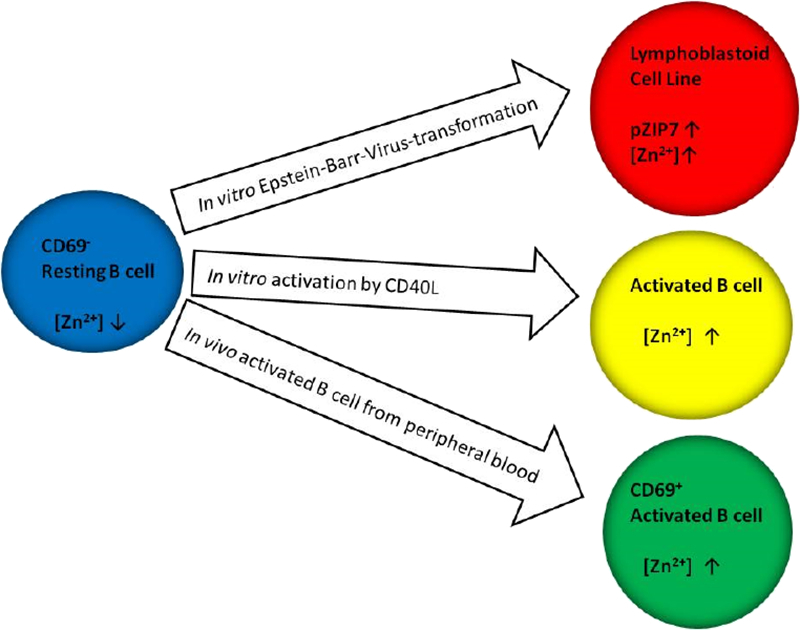

## 1 Introduction

Zinc is an essential trace element and fulfills numerous functions in the human body. Since zinc deficiency was confirmed in 1963 to cause severe effects in humans [1], various researchers have centered their interest on the study of zinc and its effects.

The importance of zinc is described especially for the immune system [2]. Both, innate and adaptive immunity, rely on the accurate concentration of zinc and regulation of zinc transporters to ensure zinc homeostasis [3]. Zinc deficiency severely affects the immune system, as impressively shown in acrodermatitis enteropathica, an inherited disorder with a loss-of-function mutation of ZIP4 which is accompanied by zinc deficiency [4]. In this study, we examined B cells, whose correct function is indispensable for the human immune system. B cells are antigen-presenting cells, which produce antibodies and cytokines, represent the immunological memory and even seem to have regulatory and suppressing functions in inflammation [5]. In mice, dietary zinc deficiency leads to lymphopenia by loss of precursor B cells [6]. In addition, zinc deficiency reduces T cell-dependent antibody responses of B cells [4]. Thus, apart from the better-known effects of zinc on T cell functions, the B cell system is also affected by zinc deficiency.

The total amount of zinc in a human body is about 2–3 g with the highest concentrations in bone, prostate and pancreatic tissue [7]. Many physiological functions are dependent on zinc, because it performs catalytical and structural roles within enzymes [8]. More than 3000 proteins are estimated to have a zinc binding motif, including metalloenzymes and transcription factors like zinc finger proteins [9]. These proteins buffer most intracellular zinc with high affinity [10]. However, a significantly smaller pool of zinc ions exists in a “free” or “labile” intracellular form [11]. In this manuscript, the term “free” will be used for these ions. In fact, the free zinc can also be bound slightly to organic and inorganic low molecular weight molecules [12]. The concentration of zinc ions in this pool ranges from high picomolar to low nanomolar [13]. Free zinc ions can induce effects in cells as second messengers in various signaling pathways [2], [14]. Hence, a strict control of homeostasis and fluctuations of the small zinc pool is necessary at the cellular level to prevent unwanted signaling.

24 known zinc transporters regulate intracellular zinc levels by carrying zinc ions across biological membranes [14], [15], [16]. In detail, the zinc transporters belong to two families, 14 human Zrt-/Irt-like proteins or solute carriers 39A (ZIP/SLC39A) and 10 zinc transporters or solute carriers 30A (ZnT/SLC30A) are known so far. ZIPs are zinc importers, which transport zinc ions into the cytoplasm, either from the outside of a cell or from an intracellular compartment. ZnTs, in contrast, are exporters, which facilitate zinc efflux out of the cell and into intracellular organelles and storage systems [17]. Current knowledge of structure, localization and function of the several zinc transporters has been reviewed in detail elsewhere [14], [15], [16], [17].

Apart from other effects, zinc ions promote proliferation *via* major protein kinase pathways such as the phosphatidylinositide 3-kinase (PI3K)/AKT cascade or the extracellular signal-regulated kinase (ERK) pathway [18], [19], [20], especially downstream of a ZIP7-mediated zinc store release [21]. Cell proliferation is pivotal, but uncontrolled proliferation, *e.g. via* hyperactivation of these proliferation-promoting signaling molecules, leads to great damage and is a common phenomenon in cancer [22]. In recent years, altered intracellular zinc homeostasis and expression of zinc transporters has been found in various cancer cells [23], [24], [25]. Common characteristics of malignant cells are abnormal regulation processes, resulting in autonomous growth, increased lifespan [26] and suppression of apoptosis [27]. Suppressed apoptosis allows mutated cells to survive, to accumulate mutations without being eliminated and to gain malignancy [27]. In addition to its role in proliferation pathways, zinc inhibits pro-apoptotic enzymes like caspases and, thus, prevents apoptosis [28], [29], [30]. However, both, very high and low zinc levels, can initiate cell death *via* different pathways [31], [32].

For example, higher zinc supply by overexpression of the zinc importer ZIP4 in pancreatic cancer cells is suggested to be associated with higher proliferation rates and tumor progression *via* phosphorylation of a zinc finger transcription factor and subsequent acceleration of the cell cycle creating higher cell proliferation [33]. Moreover, this zinc importer seems to be involved in the regulation of matrix metalloproteinases (MMP), neuropilin-1 (NRP-1) and its ligand vascular endothelial growth factor (VEGF). These molecules are known to increase a tumor's malignancy by enhanced angiogenesis, invasion or metastasis [34].

The aim of this study was to determine the effects of proliferation and immortalization of B cells on the concentration of free intracellular zinc and expression of the 24 known zinc transporters at the RNA level. Immortal B cells were gained by *in vitro* infection and transformation of healthy, freshly isolated B cells with EBV, thus, generating lymphoblastoid cell lines (LCL). In their lymphoblastoid form, B cells are known to be larger and activated, which is consistent with the production of certain viral products and rapid proliferation [35]. However, EBV does not only transform B cells into immortal lymphoblastoid cells *in vitro* but it is also associated with cancer development *in vivo*. Hodgkin lymphomas, some non-Hodgkin lymphomas, comprising the endemic Burkitt lymphoma, and tumors, like the nasopharyngeal carcinoma and some gastric carcinomas, are all known to be partially associated with EBV infection [36].

An increased intracellular free zinc level was found in the LCL, consistent with overexpression of ZIP7 RNA and protein. Furthermore, an increased phosphorylation of ZIP7 protein was observed, which implies an activating post-translational modification on ZIP7. Other activated B cells, like *in vitro* generated activated B cells and *in vivo* activated and freshly isolated B cells, were examined as well to exclude further influencing factors. The latter were generated by *in vitro* CD40 ligand (CD40L)-CD40 ligation. Activation was determined by the expression of the activation marker CD69.

The observed increased amounts of ZIP7 protein and its increased phosphorylation could also be confirmed in these *in vitro* generated activated B cells. Both, *in vitro* generated activated B cells and freshly isolated CD69+ B cells showed accordingly elevated levels of free zinc, which is why activation and proliferation, involving an expression of CD69 in B cells, were assumed to be the determining factors for the observed changes.

## 2 Materials and methods

### 2.1 Isolation of human peripheral blood mononuclear cells (PBMC)

Samples of lithium-heparinized peripheral venous blood of healthy donors, aged between 20 and 28 years, were diluted with equal amounts of PBS (Sigma-Aldrich, Germany) at room temperature. PBMC were isolated by density gradient centrifugation over Biocoll separating solution (Biochrom, Germany, density of 1.077 g/mL) and were collected from the interface, washed thrice with PBS and resuspended in the required medium as described below.

### 2.2 Cell culture

EBV-transformed B cells were grown in IMDM medium and Raji cells in RPMI 1640 medium (both Sigma-Aldrich, Germany). Both media were supplemented with 10% FCS (PAA, Germany), 2 mM L-glutamine, 100 U/mL penicillin and 100 μg/mL streptomycin (all from Sigma-Aldrich, Germany). The adherent murine fibroblast CD40 ligand (CD40L)-transformed NIH/3T3 cells, kindly provided by Dr. Gordon Freeman (DFCI, Boston, MA, USA), were grown in DMEM-Ham's/F12 medium supplemented with 10% FCS, 4 mM L-glutamine, 100 U/mL penicillin, 100 μg/mL streptomycin and 10 mM 4-(2-hydroxyethyl)-1-piperazineethanesulfonic acid (HEPES). All cells were cultured at 37 °C in a humidified 5% CO_2_ atmosphere.

### 2.3 EBV-transformation

1x10^7^ cells/mL of freshly isolated PBMC in RPMI 1640 medium enriched with 2% FCS were incubated for 15 min at room temperature in a 1:1 ratio with lysosomotropic L-leucine methyl ester hydrochloride G-2550 (H-Leu-Leu-OMe · HBr from Bachem, Switzerland, 0.085 mg/mL in RPMI 1640 medium) to deplete lysosome-rich cells and allow an undisturbed clonal growth of transformed B cells [37]. The cells were washed thrice with RPMI 1640 medium enriched with 2% FCS, resuspended in EBV-containing supernatant of B95–8 cells and split into portions of 1.5 mL in 6 well plates. 1.5 mL IMDM with the above-mentioned supplements were added to each well after incubation of two hours at 37 °C and 5% CO_2_. Cells were subsequently incubated overnight, collected the next day, washed and counted with trypan blue (Sigma-Aldrich, Germany) staining to adjust them to 6x10^5^ living cells/mL. When transformed cells and clusters were microscopically found, cells were split (1:1 with fresh IMDM) and grown in flasks (1x10^6^/mL). Cells were split twice per week and approximately 20 days later the following measurements were performed.

### 2.4 Activation of B cells by murine CD40 ligand transfected (tCD40L) NIH/3T3 cells

Activation of B cells was performed as previously described by Freeman et al. [38] with small modifications to the instruction by Liebig et al. [39], as FCS was used instead of FBS and human serum, and a combination of 100 U/mL penicillin and 100 μg/mL streptomycin instead of gentamycin. Human insulin (Insuman Rapid) was purchased from Sanofi (France) and human recombinant transferrin from Sigma-Aldrich (Germany).

### 2.5 Flow cytometric measurement of free intracellular zinc ions in B cells

2x10^6^ cells were loaded with the fluorescent membrane-permeable probe FluoZin3-acetoxymethyl ester (AM) (1 μM, Invitrogen, Germany) in measurement buffer [40] for 30 min at 37 °C. The membrane permeability of FluoZin3-AM is provided by the acetoxymethyl ester, whose non-polarity enables the probe to cross membranes. Once invaded in the intracellular space the ester is degraded rapidly and the probe is trapped [13]. Afterwards, cells were washed with and resuspended in 100 μL measurement buffer and stained with either 4 μL anti-CD19-phycoerythrin (PE), or anti-IgG1-PE as isotype control. 10 μL anti-CD69-allophycocyanin (APC), or anti-IgG1-APC were added for the measurement of free intracellular zinc in CD19+ activated B cells (all antibodies were purchased from BD Biosciences, Germany). After incubation for 20 min at room temperature in the dark, cells were washed again and resuspended in 1 mL measurement buffer and distributed to three tubes. Cells were incubated for 10 min at 37 °C with either no addition, 50 μM of the membrane-permeant chelator N,N,N′,N′-tetrakis(2-pyridylmethyl)ethane-1,2-diamine (TPEN) (Sigma-Aldrich, Germany) for determination of minimal fluorescence (F_min_) or combination of 100 μM ZnSO_4_ and 10 μM of the ionophore pyrithione (Sigma-Aldrich, Germany) for determination of the maximal fluorescence (F_max_). Subsequent measurements were performed with a FACSCalibur (BD Biosciences, Germany). The concentration of free intracellular zinc was calculated as described before [41] using a dissociation constant (K_D_) with 8.9 nM of the FluoZin3/Zn^2+^ complex [13].

### 2.6 Flow cytometric measurement of the activation status of B cells

1x10^6^ cells were incubated for 20 min at room temperature in the dark with either 4 μL anti-CD19-PE and 10 μL anti-CD69-APC in 100 μL PBS supplemented with 1% FCS or 4 μL anti-IgG1-PE and 10 μL anti-IgG1-APC. Cells were washed and resuspended in 500 μL PBS with 1% FCS. Fluorescence measurements were performed with FACSCalibur.

### 2.7 RNA isolation and quantitative real time polymerase chain reaction (qRT-PCR)

1x10^7^ cells were resuspended in 1 mL TRI Reagent Solution (Ambion Life Technologies, Germany) and RNA was extracted following the manufacturer's instructions. qScript cDNA Synthesis Kit (Quanta Biosciences, Germany) was used for cDNA transcription according to the manufacturer's manual. qRT-PCR was performed with a StepOnePlus Real-Time PCR System (Applied Biosystems, Germany) with 5 μL irradiated water (H_2_O), 12.5 μL SYBR Green PCR Master Mix (Applied Biosystems, Germany), 5 μL cDNA, and the respective forward and reverse primers, 1.25 μL (2 μM) each, with the following cycle program: initial denaturation for 10 min at 95 °C, 40 cycles of denaturation for 15 s at 95 °C and annealing/elongation for 30 s at 60 °C (for ZnT10 at 65 °C). Expression of ZIP1–3 [42], ZIP4–14 [43], ZnT1–9 [44], ZnT10 (forward CAC CCA GAA TGA GCC AGA AGA C and reverse GAT AAG CGG GAA GGC AGA TGA C) and porphobilinogen deaminase (PBGD) [45] was analyzed. Presented is the relative expression of RNA in LCL after EBV-transformation, normalized to the housekeeping gene PBGD. To allow a presentation of the data of transporters that were not determined before cycle 40, their expression level was set to cycle 40. The ΔΔC_T_-method was performed to compare the transporters' expression in the B cell enriched PBMC before transformation with the expression in LCL. RNA samples were measured with and without addition of H-Leu-Leu-OMe · HBr to exclude interference of this reagent with the RNA (data not shown).

### 2.8 Western blot analysis of ZIP7 protein and phosphorylated (p)ZIP7 protein

Cells were lysed by sonication using a Vibra Cell sonicator (Sonics & Materials, USA) and heated for 5 min at 95 °C. An equivalent of 3x10^6^ cells per lane was separated at 170 V on 10% polyacrylamide gels. After separation, samples were blotted onto nitrocellulose membranes (GE Healthcare Life Sciences, Germany). Uniform loading of gels was confirmed by Ponceau S (AppliChem, Germany) staining. Subsequently, membranes were blocked for 1 h with TBS-T (20 mM Tris–HCl [pH 7.6], 137 mM NaCl, 0.1% [v/v] Tween 20), containing 5% fat-free dry milk, and were washed afterwards thrice with TBS-T. Incubation with primary mouse monoclonal pZIP7 (S275/S276) [21], ZIP7 (ProteinTech, United Kingdom) and β-actin (Cell Signaling Technology, Germany) antibodies was performed overnight at 4 °C at 1/1000 dilution in TBS-T, containing 5% BSA. Afterwards, membranes were washed thrice with TBS-T and incubated with either anti-rabbit-HRP (for β-actin and ZIP7) or anti-mouse-HRP (for pZIP7) (both from Cell Signaling Technology, Germany) secondary antibodies 1/2000 in TBS-T with 5% fat-free dry milk. After again washing thrice with TBS-T, immunodetection was performed using LumiGlo Reagent (Cell Signaling Technology, Germany) on LAS-3000 (Fujifilm Lifescience, Germany). Band density was determined with ImageJ software (NIH, USA).

### 2.9 Statistical analysis

Statistical analyses were performed with GraphPad Prism software (La Jolla, USA). The Student's t-test and repeated measurement ANOVA with Bonferroni's multiple comparison test were applied.

## 3 Results

### 3.1 Increased free zinc levels were found in human B cells upon EBV-transformation

**Fig. 1:**
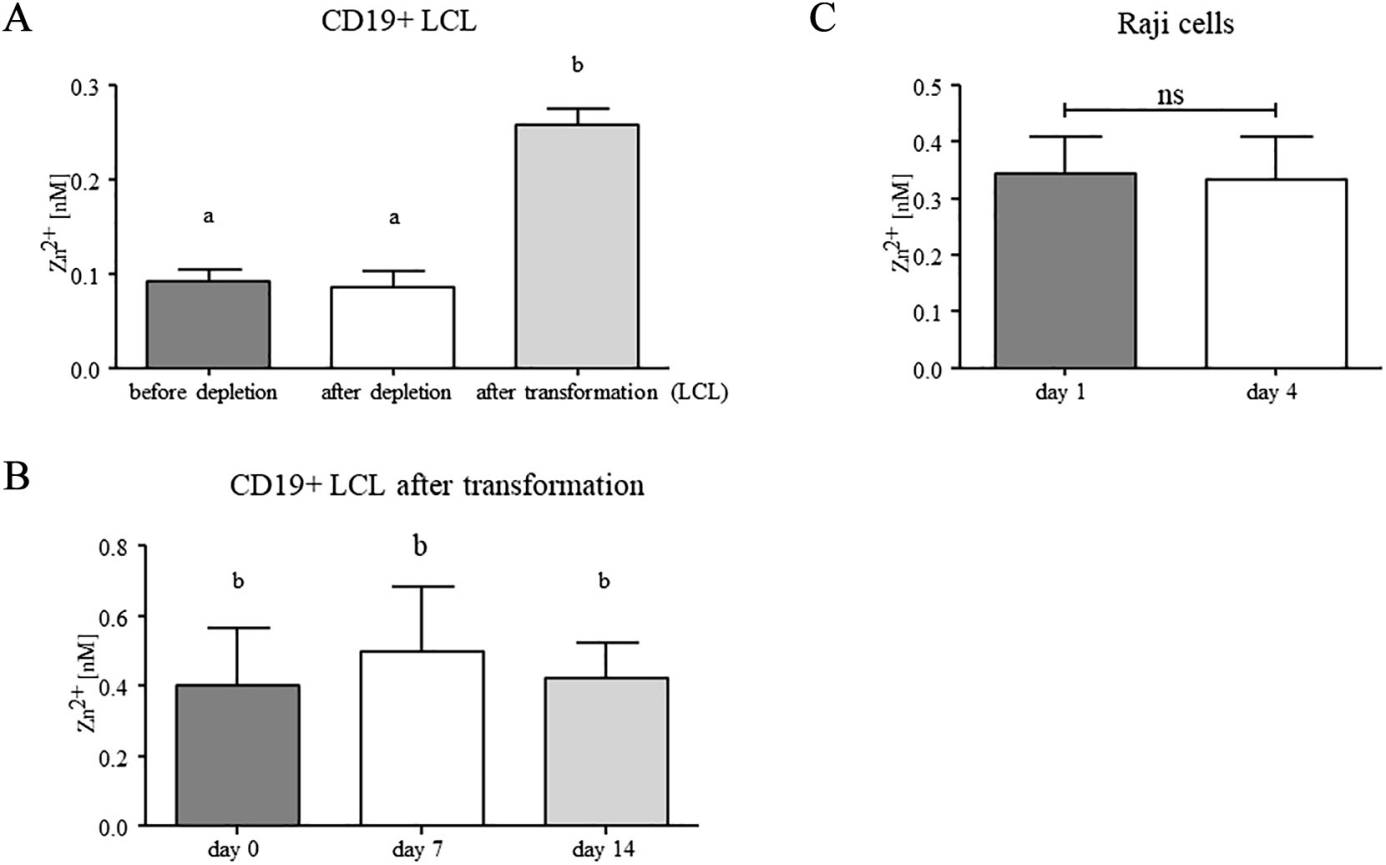
**Intracellular zinc concentration in B cells before and after transformation and in Raji cells.** Measurements were performed in freshly isolated CD19+ PBMC before depletion of T cells, CD19+ PBMC after T cell depletion and in CD19+ transformed B cells (LCL). No difference between the intracellular zinc concentration before and after T cell depletion was seen. The LCL show significantly higher intracellular zinc levels than non-transformed B cells (A). The intracellular free zinc was repeatedly measured in LCL, starting at different time points after transformation (day 0) and following up at day 7 and day 14 to prove whether the elevation of the zinc level would endure. The first control was performed with *n* = 3, 20 to 94 days after transformation. The second and third controls were performed each one week later (B). The concentration of free intracellular zinc in Raji cells did not significantly change between the first and the fourth day after splitting of the cells and addition of fresh medium (C). Mean values + standard error of the mean (SEM) of *n* = 15 (A), *n* = 3 (B, C) are presented. Means sharing the same letter are not significantly different from each other (*p* < 0.05, repeated measurements ANOVA and Bonferroni's multiple comparison test for A and B). Nonsignificant differences between means are marked with ns (*p* > 0.05, Student’s t test for C).

*In vitro* EBV-infection of B cells gives rise to lymphoblastoid cells, which is called transformation or immortalization. CD19 is a surface protein that is expressed exclusively on the cell surface of the B cells and co-ligates with the B cell receptor [46]. In this study CD19 was used to identify B cells. The concentration of free zinc in CD19+ cells was significantly increased in the LCL compared to non-transformed B cells (Fig. 1A). The free zinc level in PBMC was not affected by H-Leu-Leu-OMe · HBr, which was used for T cell depletion (Fig. 1A). The elevated zinc level in the LCL did not decrease during long term culturing, as repeated control measurements confirmed (Fig. 1B). To exclude culture conditions as a reason of changes in intracellular zinc levels, the zinc concentration was measured in Raji cells at different timepoints after subculture (Fig. 1C). In 1963, the Raji B cell line was derived from an African EBV+ Burkitt lymphoma [47] and served as an adequate control cell line in this study. The cells did not alter their intracellular zinc concentrations during culture time. Therefore, the observed changes of intracellular zinc levels in transformed B cells are unlikely attributed to culture conditions.

This data together suggests that intracellular free zinc levels are influenced by EBV-transformation/immortalization of B cells.

### 3.2 Activated B cells show higher amounts of intracellular free zinc

To understand whether elevated zinc levels rely on the immortalization or rather the activation of cells, the free zinc concentration in *in vitro* activated B cells was determined in a subsequent experiment. To generate these activated B cells in the absence of EBV, an *in vitro* simulation of the T cell mediated B cell activation by CD40-CD40 ligand (CD40L) ligation and pathway induction was applied. *In vivo*, CD40L is presented by activated T cells and ligation with CD40, a tumor necrosis factor expressed by B cells, leads to activation of signaling pathways, including protein tyrosine kinase activation [48]. PBMC were incubated with murine tCD40L NIH/3 T3 cells in the presence of interleukin (IL)-4 and ciclosporin A (CsA) [38], [39].

**Fig. 2:**
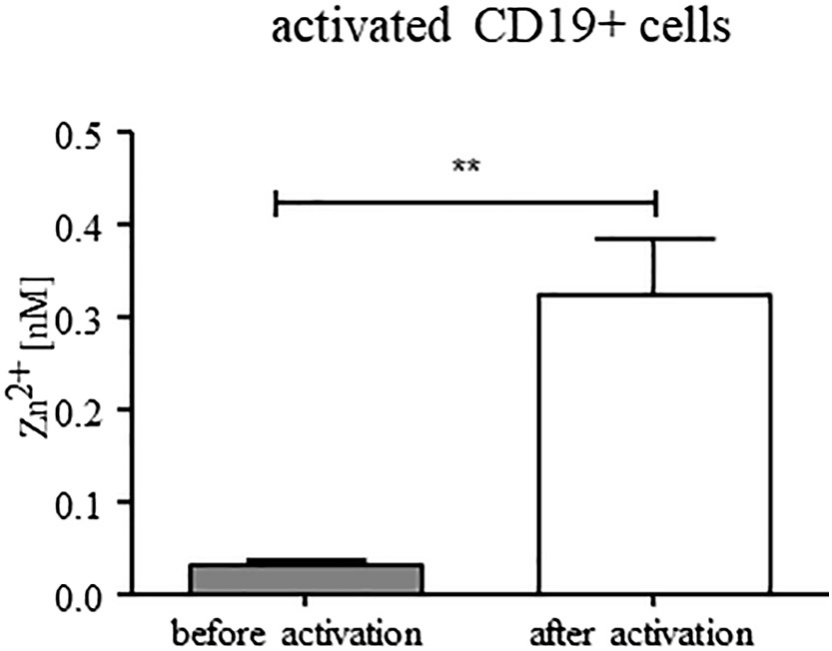
***In vitro* activation of B cells positively influences the level of free intracellular zinc.** Free intracellular zinc was measured after dyeing with FluoZin3-AM and CD19-PE. Activated B cells were generated from PBMC through stimulation by tCD40L/NIH3T3 cells, IL-4 (50 U/mL) and CsA (0.63 µg/mL). After activation, CD19+ cells showed a significant higher level of free intracellular zinc. Presented is the mean + SEM of *n* = 7 (** *p* < 0.01; Student’s t-test).

To assess the activation state, the expression level of the surface marker and activation inducer molecule CD69 was determined. It is expressed on activated lymphocytes, including LCL. After crosslinking, CD69 is involved in the response of lymphocytes to various stimuli, including the proliferative response. Resting B cells do not show CD69 surface expression [49], [50]. Both, the CD40L-activated B cells (about 16.5% CD69+) and the cultured CD19+ LCL (about 50% CD69+), showed high levels of CD69 on their surface, whereas this marker was hardly expressed in non-activated PBMC (data not shown) [51], [52]. The intracellular free zinc levels in CD40L-activated B cells were comparably elevated to that of CD19+ LCL with a mean of 0.324 nM (+ SEM 0.060 nM) (Fig. 2).

In summary, this data suggests activation or proliferation of B cells as more likely factors for the observed increased zinc levels than immortalization of B cells.

### 3.3 Increased CD69 marker expression is associated with increased intracellular zinc levels

**Fig. 3:**
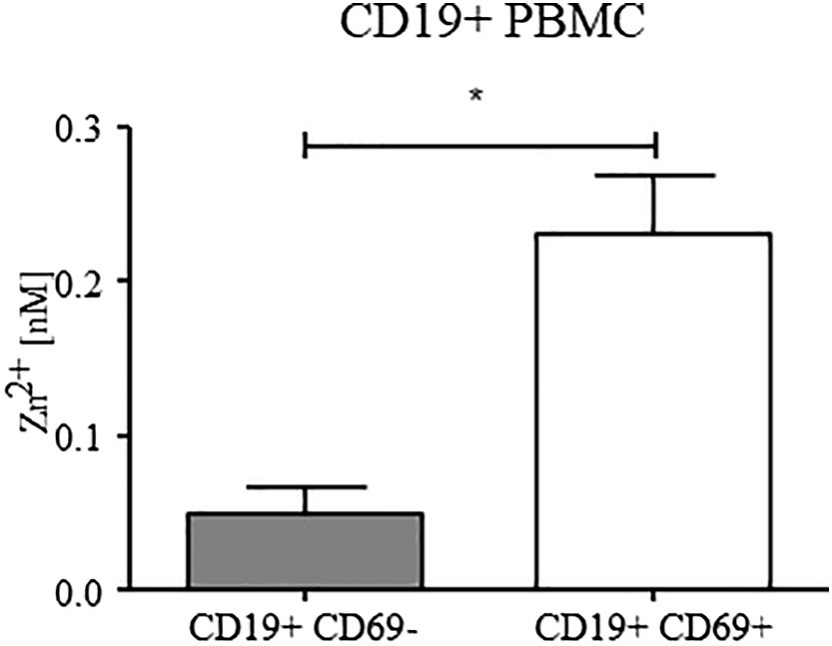
**Expression of the activation marker CD69 is associated with a higher level of free intracellular zinc in B cells.** Measurements were performed in freshly isolated CD19+ PBMC. Within those, CD69+ showed significant higher intracellular zinc levels than CD69- cells. Presented is the mean + SEM of *n* = 3 (* *p* < 0.05, Student’s t-test).

So far, analyses were performed in EBV-immortalized and *in vitro*-activated B cells. In the following experiment, the concentration of free intracellular zinc and the expression of the activation marker CD69 in freshly isolated CD19+ PBMC was determined. The zinc concentrations in CD69+ and CD69- subpopulations were compared (Fig. 3) in order to exclude other possible influencing factors associated with the necessary treatment of B cells and the following culturing conditions. The free intracellular zinc level in CD69+ B cells was enhanced, comparable to the concentrations in LCL and activated B cells, while the CD69- cells showed lower levels of free intracellular zinc.

### 3.4 Zinc transporter expression is changed in transformed and activated human B cells

**Fig. 4:**
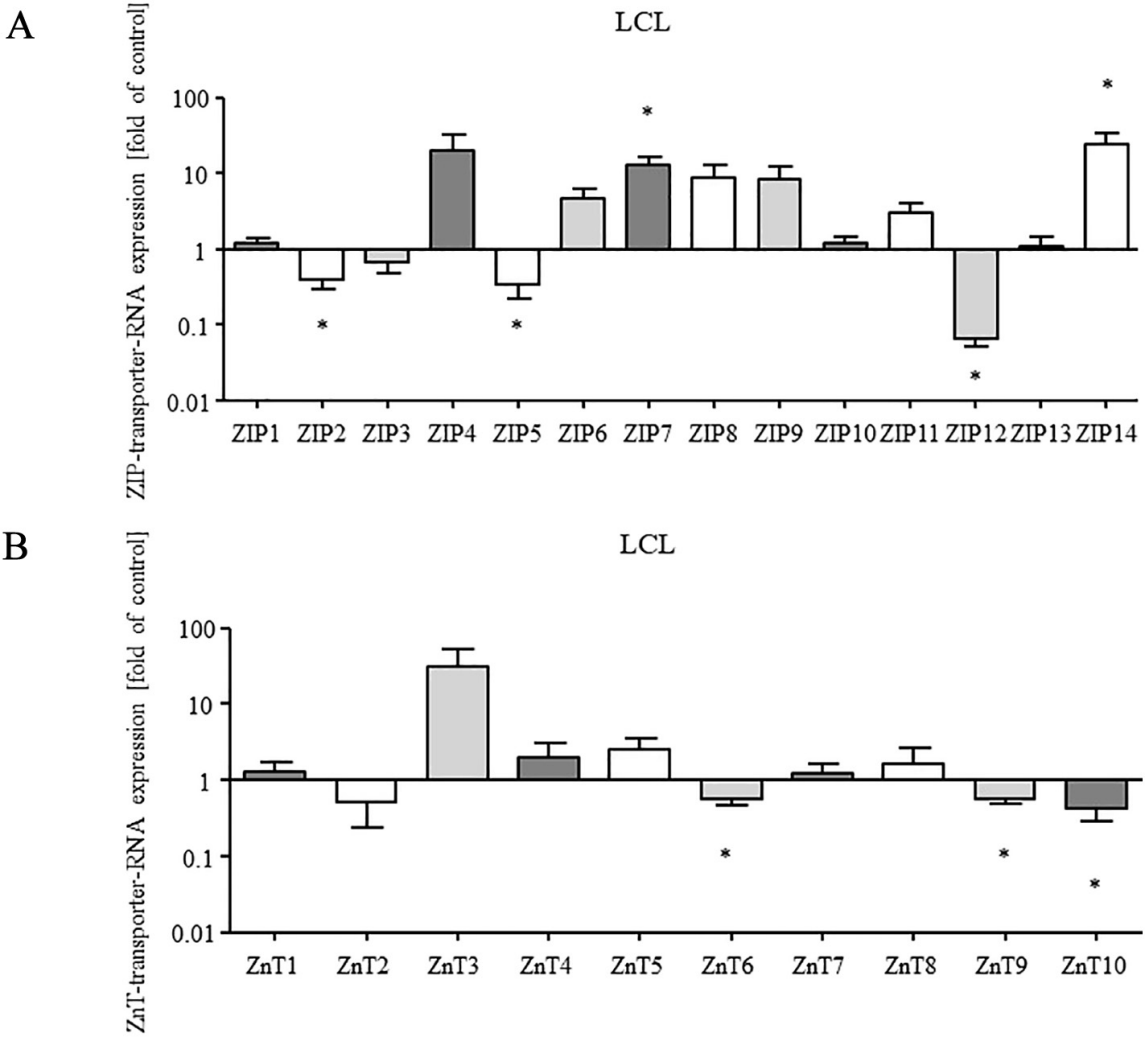
**RNA expression of several zinc transporters is altered after EBV-transformation.** The RNA expression in LCL was compared to T cell depleted PBMC. The ΔΔC_T_-method was used for quantification. Cycle 40 was set, when transporters were non-expressed before or after transformation. Shown is the relative expression of RNA after EBV-transformation in comparison with the expression of RNA in T cell depleted PBMC, equaling a relative expression of 1, normalized to the housekeeping gene PBGD. Mean values + SEM of *n* = 9 (A) and *n* = 7 (B) are shown (* *p* < 0.05, Student’s t-test).

Since the levels of intracellular zinc were increased in transformed B cells we further studied the expression of zinc transporters on RNA levels to find possible associated alterations (Fig. 4).

The relative RNA expression of various zinc transporters was found to be altered in the LCL compared to that of T cell depleted PBMC. Expression of ZIP importers is shown in Fig. 4A and ZnT exporter expression is presented in Fig. 4B. The lysosomotropic agent applied for T cell depletion did not itself interfere with the RNA quantification. Hence, the treated PBMC could be used for normalization. The LCL significantly upregulated the zinc importers ZIP7 and ZIP14 (Fig. 4A) on RNA levels compared to the T cell depleted PBMC, whereas the expression of ZIP2, ZIP5 and ZIP12 (Fig. 4A) was significantly reduced as well as expression of ZnT6, ZnT9 and ZnT10 RNA (Fig. 4B). Variations in expression levels are high due to the use of individual human samples. Therefore, the expression of zinc transporters is not always significant, as one might expect, and indicated by the respective significancies (Fig. 4).

To confirm whether the altered expression was caused by transformation or by culturing conditions, transporter expression was also analyzed in Raji cell culture. A decrease of ZIP3 RNA could be detected 24 h and 72 h after subculturing in fresh medium, whereas, the expression of ZnT3 RNA was decreased 72 h after subculturing. Although a significant increase of RNA expression following time of culture could not be detected, an impact of culturing conditions on the expression of the mentioned transporters cannot be excluded (data not shown).

**Fig. 5:**
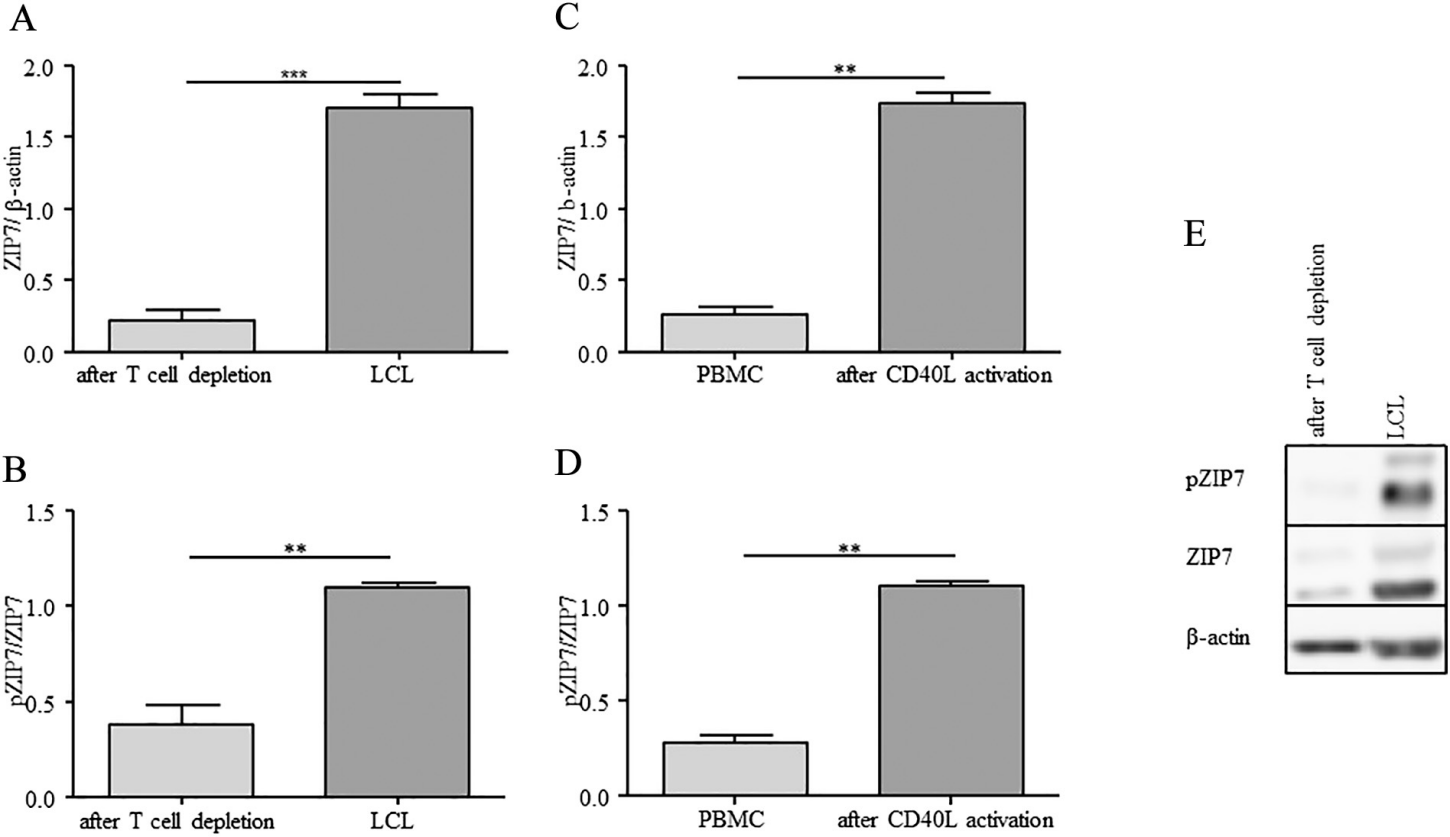
**ZIP7 and phosphorylation of ZIP7 (pZIP7) are increased after immortalization and activation.** Analysis of ZIP7 expression in LCL (A) and CD40L-activated B cells from freshly isolated PBMC (C) showed increased ZIP7 protein levels compared to measurements in only T cell depleted PBMC or freshly isolated PBMC, respectively. Apart from that, not only the amount of ZIP7 was increased, but also phosphorylation of the ZIP7 importer (B, D). (A-D) Densitometric quantification of *n* = 5 (A, B) and *n* = 3 (C, D) is presented as mean + SEM (** *p* < 0.01, *** *p* < 0.001; Student’s t-test). (E) Immunodetection of one representative Western Blot.

Consistent with the findings on RNA levels in LCL, elevated levels of ZIP7 on the protein level were found in both, LCL (Fig. 5A) and in *in vitro* activated B cells (Fig. 5C). Furthermore, an increased phosphorylation of ZIP7 protein (pZIP7) could be demonstrated in immortalized and in CD40L-activated B cells (Fig. 5B, D).

## 4 Discussion

All in all, the data suggest that activation and proliferation of B cells, rather than other factors like immortalization or long-time culturing, is associated with increased intracellular zinc concentrations. High proliferation rates are also a common characteristic of tumor cells. Thus, it seems reasonable, that the highly proliferative CD69+ B cells share some traits with the latter. Actually, alterations of zinc transporters going along with increased zinc levels were previously found in various solid cancer cells, *e.g.* in invasive breast carcinoma [24] and malignant ovarian tumors [53]. Inconsistent studies indicate at least an effect of malignancy on the zinc level in pancreatic cancer, as they show either elevated [25] or decreased zinc levels [54]. Prostate carcinoma cells have been well studied regarding their zinc homeostasis and they are an outstanding exception. They downregulate their ZIP4 expression [55] and show decreased concentrations of zinc [56]. This discrepancy to the behavior of other tumors might be due to the fact that zinc levels in normal prostate tissue exceed those of other soft tissues with 1.018±0.124 mg zinc/g dry tissue [57]. Numerous studies also indicate an association between the aberrant expression of certain zinc transporters and malignancy in tissue samples from cancers and cell lines. The higher intracellular zinc levels might result from an increased expression of zinc importers and serve a fast-growing tumor cell to maintain sufficient zinc for further proliferation [25]. The observed changes in zinc homeostasis and transporter expression in cancer cells and in LCL might have similar reasons and functions. LCLs encode various viral products, amongst these the latent membrane protein 1 (LMP1) [35]. LMP1 is an oncogene and seems to mimic CD40-CD40L-interaction [58]. On the molecular level, both LMP1 and the CD40-signaling activate the nuclear factor kappa-light-chain-enhancer of activated B cells (NF-κB) [59], [60]. The activated NF-κB is an important signaling molecule, especially in the immune system, inducing several anti-apoptotic proteins, stimulating cell cycle progression and playing a crucial role in carcinogenesis [61]. Its either constitutive expression or over-expression was demonstrated in various cancers, including solid and hematopoietic cancers [61]. Moreover, NF-κB induces the expression of CD69 [51], which is expressed at the surface of activated, proliferating lymphocytes [62]. Activation and proliferation of B cells are crucial for a functional immune system. Physiologically, B cells need the CD40-CD40L-interaction in addition to further signals to become activated and to start proliferation. CD40 is a B cell surface protein, while CD40L is physiologically presented by activated T cells [63].

In the current study, we utilized CD40L-transfected murine fibroblasts in combination with IL-4 in the absence of T cells to generate an adequate T cell independent growth signal for B cells [38]. Both, the LCL and the CD40L-activated B cells, expressed high levels of CD69 on their surface. Within these cells, the level of free zinc was found to be elevated. Levels of free zinc were also higher in freshly isolated PBMC, expressing the surface markers CD19 and CD69, compared to zinc levels found in CD19+ and CD69- cells. We therefore suggest an association between the expression of the CD69 activation marker on the cell surface of CD19+ B cells and an elevated intracellular zinc level. The elevated zinc levels observed in LCL and CD40L-activated B cells in the present study are likely originating from the endoplasmatic recticulum (ER) upon activation, due to the detected increased amounts of ZIP7 increased phosphorylation of ZIP7 after activation, which is especially found with the membrane of the ER [64]. The elevated free zinc might serve as a co-stimulus for the elevation of NF-κB due to LMP1 or CD40-signaling. Another explanation might be that the NF-κB pathway leads to increased free intracellular zinc by alternating the expression of zinc transporters and thus gaining malignant cellular characteristics.

With respect to signaling pathways, zinc has already been shown to stimulate proliferation *via* the PI3K/AKT cascade and/or ERK, *e.g.* in T cells [18] and murine myogenic cells [19]. Both molecules, AKT and ERK, are serine/threonine kinases. AKT is known to prevent apoptosis, promote cell survival and seems to raise cell cycle progression by its manifold downstream targets [65]. ERK is involved in the mitogen-activated protein kinase (MAPK)/ERK pathway and stimulates cell proliferation and survival when phosphorylated [19], [66]. These kinases regulate the cell cycle *via* phosphorylation of downstream molecules. Aberrations that affect these pathways, especially constitutive activation, are known to be involved in the development of a wide range of cancers [67]. We suggest a comparable upregulation of cell cycle regulating pathways in CD69+ B cells, matching the observed increase of free intracellular zinc.

Having demonstrated an increase of free intracellular zinc in the activated and proliferating cells, we also showed an elevation of the RNA level of zinc importers ZIP7 and ZIP14 in LCL. ZIP7 protein is known to play an important role in zinc release and its phosphorylation activates proliferation pathways in human cells [21]. There exist antibodies for both, ZIP7 and phosphorylated ZIP7, therefore ZIP7 was chosen in this study for closer analysis on protein levels. However, determination of other transporter expression on protein levels might be interesting for upcoming studies. An increase of ZIP7 protein was confirmed in both, LCL and CD40L-activated B cells. Not only was the total amount of ZIP7 protein increased after transformation, but also its phosphorylation, which indicates increased activation of the transporter. The post-translational phosphorylation of the ZIP7 transporter is proposed to be crucial for the release of stored zinc and the generation of intracellular zinc waves [68]. ZIP7 protein is localized on inner membranes, such as the membrane of the endoplasmic reticulum [15] and of the Golgi apparatus [69]. It enables the release of stored zinc to prevent cytoplasmic zinc deficiency as well as an accumulation in the Golgi apparatus [69]. The increase of free zinc, following a ZIP7-mediated zinc release, inhibits phosphatases and therefore augments signaling by less deactivation and subsequent accumulation of phosphorylated kinases such as ERK [64] and AKT [21].

An increase of both ZIP7 RNA and protein levels has been shown by Taylor et al. in aggressive tamoxifen resistant breast cancer cells, compared to the non-resistant cells, thereby demonstrating activation of cancer promoting pathways by the increase of zinc, leading to proliferation and aggressive growth [24].

Related observations of the signaling in physiological conditions were made in chicken DT40 B cells. Here, ZIP9 led to zinc release from intracellular stores after B cell receptor (BCR)-cross linking. Since DT40 cells do not express ZIP7, ZIP9 might replace the functions that ZIP7 fulfills in human cells. The authors propose the zinc wave to inhibit tyrosine phosphatases, *e.g.* by degradation or by binding to an active site, and therefore to prevent deactivation of phosphorylated ERK and AKT and to lead to prolonged signaling [70].

Furthermore, in the present study ZIP14 RNA was elevated in LCL. ZIP14 overexpression has already been shown to increase the proliferation of hepatocytes in mice, again by phosphorylation of kinases [71].

Even though there are differences between breast cancer cells, chicken B cells, proliferating hepatocytes and the studied B cells, some similarities regarding the affected pathways can be suggested. Therefore, we assume our findings to be part of the signaling pathways, including a phosphorylation of kinases, to enable high proliferation and activation rates of cells. The upregulation or increased activation of zinc importers might explain the observed elevation of free intracellular zinc in *in vitro* EBV-transformed B cells, which in turn might lead to increased signaling within the cells. These findings suggest upregulation of zinc importers, particularly ZIP7 upregulation, as a common phenomenon in B cells with a high proliferation rate, playing an important role in the signaling activities of a cell.

Additionally, a decrease of RNA expression of the zinc importers ZIP5 and ZIP12, but also of the zinc exporters ZnT6, ZnT9 and ZnT10 was found in the present study. The latter might ensure high intracellular zinc levels due to lower zinc export.

In summary, significantly elevated zinc levels have been shown in activated B cells. Furthermore, in the LCL, elevated zinc levels are accompanied by altered zinc transporter expression at the RNA level. A relation between increased zinc levels and the highly expressed activation marker CD69 has also been demonstrated. Moreover, not only was there observed upregulation of ZIP7 RNA there was also increased ZIP7 protein levels in LCL and CD40L-activated B cells, being accompanied by increased ZIP7 phosphorylation, consistent with active release of zinc from stores.

In conclusion, increased intracellular zinc seems to be a prerequisite of B cell activation.

## Funding

The generation of the pZIP7 antibody was funded by a Wellcome Trust University Research Award (091991/Z/10/Z) to KMT.
